# Dynamics of land use and land cover change (LULCC) using geospatial techniques: a case study of Islamabad Pakistan

**DOI:** 10.1186/s40064-016-2414-z

**Published:** 2016-06-21

**Authors:** Zahra Hassan, Rabia Shabbir, Sheikh Saeed Ahmad, Amir Haider Malik, Neelam Aziz, Amna Butt, Summra Erum

**Affiliations:** Department of Environmental Sciences, Fatima Jinnah Women University, Rawalpindi, Pakistan; Department of Environmental Sciences, COMSATS Institute of Information Technology, Abbottabad, Pakistan

**Keywords:** Land use/land cover, Change detection, GIS, Remote sensing, Islamabad

## Abstract

One of the detailed and useful ways to develop land use classification maps is use of geospatial techniques such as remote sensing and Geographic Information System (GIS). It vastly improves the selection of areas designated as agricultural, industrial and/or urban sector of a region. In Islamabad city and its surroundings, change in land use has been observed and new developments (agriculture, commercial, industrial and urban) are emerging every day. Thus, the rationale of this study was to evaluate land use/cover changes in Islamabad from 1992 to 2012. Quantification of spatial and temporal dynamics of land use/cover changes was accomplished by using two satellite images, and classifying them via supervised classification algorithm and finally applying post-classification change detection technique in GIS. The increase was observed in agricultural area, built-up area and water body from 1992 to 2012. On the other hand forest and barren area followed a declining trend. The driving force behind this change was economic development, climate change and population growth. Rapid urbanization and deforestation resulted in a wide range of environmental impacts, including degraded habitat quality.

## Background

The modification of Earth’s terrestrial surface by human activities is commonly known as Land use/land cover change (LULCC) around the globe. Although modification of land by humans to obtain livelihoods and other essentials has been there for thousands of years, the extent, intensity and rate of LULCC are far greater now than were in the past. These changes are driving forces for local, regional and global level unprecedented changes in ecosystems and environmental processes. Thus, LULC changes play an important role in the study and analysis of global changed scenario today as the data available on such changes is essential for providing critical input to decision-making of ecological management and environmental planning for future (Zhao et al. 2004; Dwivedi et al. 2005; Erle and Pontius 2007; Fan et al. 2007).

Empirical studies by researchers from diverse disciplines found that changes in land use/land cover has become key to many diverse applications such as agriculture, environment, ecology, forestry, geology and hydrology (Weng 2001). These applications referred to crop land loss, soil degradation, urban expansion, water quality change etc. At the same time, according to Lambin (1997) in the past decades, a major project to study land use change has emerged as an international initiative and has gained great impetus in its efforts to understand forces driving land use change. These efforts have stimulated the interest of researchers to apply various techniques to detect and further model environmental dynamics at different levels.

Change detection has emerged as a significant process in managing and monitoring natural resources and urban development mainly due to provision of quantitative analysis of the spatial distribution of the population of interest. There are a lot of available techniques that serve purpose of detecting and recording differences and might also be attributable to change (Singh 1989; Yuan et al. 1999). Though, simple change detection is seldom adequate in itself: there is a requirement of information regarding initial and final land cover/types/uses, the ‘‘from-to’’ analysis. It is necessary to have accurate and up-to-date land cover change information for understanding and assessment of the environmental consequences of such changes (Giri et al. 2005).

Satellite Remote Sensing and GIS are the most common methods for quantification, mapping and detection of patterns of LULCC because of their accurate geo-referencing procedures, digital format suitable for computer processing and repetitive data acquisition (Lu et al. 2004; Chen et al. 2005; Nuñez et al. 2008; Rahman et al. 2011). Digital change detection process has been used widely for determination and/or description of the prevalent changes in LULC properties based on multi-temporal remotely sensed data. The basic purpose of using this data for detecting change is its ability to identify uncharacteristic change between two or more dates. Several researchers have addressed the problem of accurate monitoring of LULCC in numerous environments (Singh 1989; Muchoney and Haack 1994; Chan et al. 2001). The land cover and land use changes in agriculturally productive, arid and semi-arid land have been discussed in many studies. Rembold et al. (2000) used Landsat TM dataset for years 1972 and 1994 to calculate land cover changes in lake regions of central/south Ethiopia. Shalaby and Tateishi (2007) identified types of LULC in the coastal zone of Egypt by making use of Landsat data. Gao and Liu (2010) performed digital analysis of two Landsat images to detect land degradation trend that occurred at an interval of 10 years due to soil salinization and waterlogging in northeast China.

Numerous techniques have been developed and used for change detection to monitor changes in LULC by making use of remotely sensed data for instance image differencing, post-classification comparison (PCC), vegetation index differencing and principle components analysis (PCA) (Lu et al. 2004). Among these, post-classification comparison was found to be the most accurate procedure by variety of studies as it offered an advantage of representation of nature of occurring changes. It compares classifications of images from different dates, which are independently produced in order to detect land cover changes (Yuan et al. 1999). Thus, making use of PCC method minimizes associated problems with multi-temporal images that are recorded under different atmospheric and environmental conditions. It separately classifies the data from different dates, and thus, this multi-date data does not require any adjustment for direct comparison (Singh 1989; Rivera 2005; Zhou et al. 2008; Warner and Campagna 2009). An additional advantage of PCC method is the indication of the nature of change (Yuan et al. 2005).

**Fig. 1:**
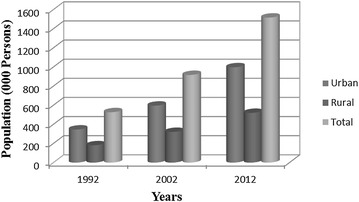
Demographics of Islamabad from 1992 to 2012

Out of many, one key problem for developing countries like Pakistan is the haphazard urban development mostly without proper planning strategies. There are number of ways in which urban growth affects the ecology of cities, such as modifying local climate conditions, eliminating and fragmenting native habitats, and generating anthropogenic pollutants. It is documented that the ecological processes are affected by spatial pattern of a landscape (Mandelas et al. 2007; Guru and Anubhooti 2015). As a consequence of population growth (natural and immigration) some rapid development outcropping usually takes place and valuable agricultural land is being encroached. Islamabad, the capital city of Pakistan in the terms of population is the country’s most diverse metropolis. It has the largest expatriate and foreigner population in the Pakistan. Constant increase in population in the capital has resulted in urban sprawl to the fertile rural land of the capital. Figure 1 presents the demographics of Islamabad from 1992 to 2012.

The urbanization process has led to chaotic growth in city, deteriorated the living conditions and has worsened the environmental scenario having detrimental impacts on human health. Therefore, it is required to determine the rate and trend of land cover/use conversion for devising a rational land use policy.

The study aimed at carrying comparative study/analysis of the LULCC of Islamabad using RS and GIS tools. This aim was achieved through the following objectives (1) to identify and delineate different LULC categories and pattern of land use change in Islamabad from 1992 to 2012 (2) to integrate supervised classification and visual interpretation using GIS and to examine the potential of integrating GIS with RS in studying the spatial distribution of different LULC changes and (3) to identify major driving forces and extent of contribution in LULC change.

### Study area

**Fig. 2:**
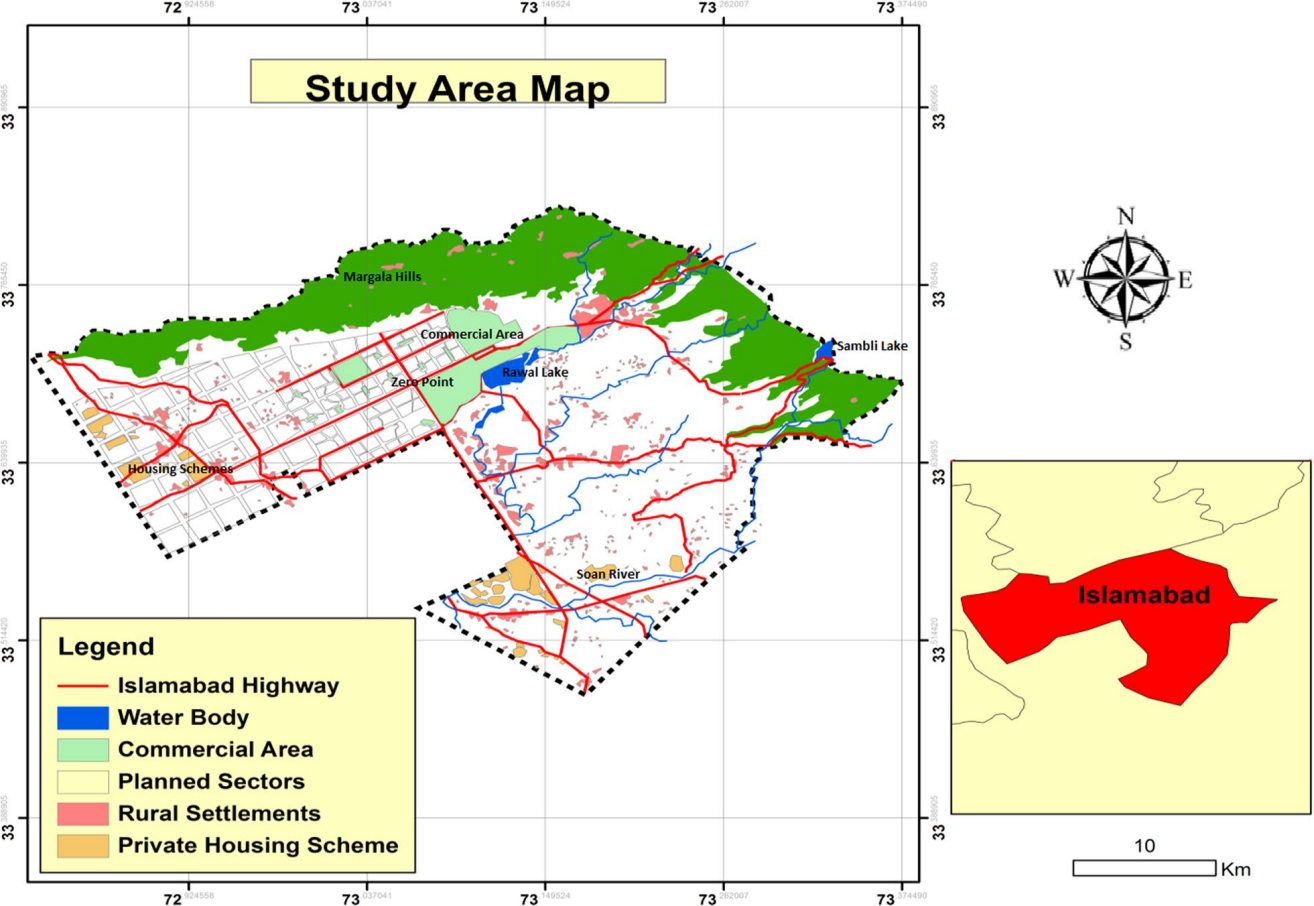
Location map of the study area

Islamabad is located 14 kms north east of Rawalpindi, on the north eastern fringe of the Potohar plateau of the province of Punjab. In terms of map reference, it is lying at 33°49′ north and 72°24′ east of Greenwich. Islamabad lies at an altitude range of 457–610 m. The area of Islamabad is 906.50 km^2^. A further 3626 km^2^ area is known as the Specified Green Area, with the Margalla Hills in the north and northeast (Fig. 2).

## Methods

### Data acquisition

In order to study LULC changes in a city like Islamabad, two multispectral satellite images of city were acquired for two Epochs; 1992 and 2012. 1992 and 2012 images (LANDSAT) were obtained for the month of October from United States Geological Survey (USGS) (http://glovis.usgs.gov), an Earth Science Data Interface and SUPARCO respectively. Specifications of the satellite data acquired for change analysis are given in Table 1. In addition to using high-resolution imagery, ancillary data was collected which included ground truth data, aerial photographs and topographic maps. The ground truth data was in the form of reference data points collected using Geographical Positioning System (GPS) from March to October 2012 for 2012 image analysis, used for image classification and accuracy assessment of the results.

**Table 1:** Satellite data specifications

Data	Year of acquisition	Bands/color	Resolution (m)	Spectral resolution/bands	Source
Landsat 5 TM imagery	1992	Multi-spectral	30	Band 2 (green) 520–600 nm	USGS glovis
				Band 3 (red) 630–690 nm	
				Band 4 (near IR) 760–900 nm	
SPOT 5 imagery	2012	Multi-spectral	10	Band 2 (green) 500–590 nm	SUPARCO
				Band 3 (red) 610–680 nm	
				Band 4 (near IR) 780–890 nm	

### Image pre-processing and classification

Satellite image pre-processing before change detection phenomenon is very important in order to establish a more direct affiliation between the acquired data and biophysical phenomena (Abd El-Kawya et al. 2011). Due to acquisition system and platform movements, remotely-sensed data from aircrafts or satellites are generally geometrically distorted. The satellite data were imported into ERDAS 2011 software in an image format for geometric correction. After the images were geo-referenced, mosaicked and subset on the basis of Area of Interest (AOI). All satellite data were studied by assigning per-pixel signatures and differentiating the land area into five classes on the bases of the specific Digital Number (DN) value of different landscape elements. The delineated classes were Built-up area, Agriculture, Forest, Water and Barren area (Table 2). Each class was given unique identity and assigned a particular colour to make them separate from each other. For each of the predetermined land cover/use type, training samples were selected by delimiting polygons around representative sites. Spectral signatures for the respective land cover types derived from the satellite imagery were recorded by using the pixels enclosed by these polygons. A satisfactory spectral signature is the one ensuring that there is ‘minimal confusion’ among the land covers to be mapped (Gao and Liu 2010). After that supervised classification was performed by applying maximum likelihood algorithm on the images. It is the type of image classification which is mainly controlled by the analyst as the analyst selects the pixels that are representative of the desired classes.

**Table 2:** Classes delineated on the basis of supervised classification

Sr. no.	Class name	Description
1	Agricultural area	Crop fields and fallow lands
2	Built up area	Residential, commercial, industrial, transportation, roads, mixed urban
3	Barren area	Land areas of exposed soil and barren area influenced by human impact
4	Forest area	Mixed forest lands
5	Water body	River, open water, lakes, ponds and reservoirs

To improve classification accuracy and reduction of misclassifications, post-classification refinement was therefore used for simplicity and effectiveness of the method (Harris and Ventura 1995). Moreover, when using a data having medium-spatial resolution such as that of Landsat mixed pixels are a common problem (Lu and Weng 2005); especially for the urban surfaces that are a heterogeneous mixture of features mainly including buildings, grass, roads, soil, trees, water (Jensen 2007). The problem of mixed pixels was addressed by visual interpretation. For the enhancement of classification accuracy and therefore the quality of the land cover/use maps produced, visual interpretation was very important. Thus, local knowledge, reference data, as well as visual analysis, considerably improved the results obtained using the supervised algorithm.

### Accuracy assessment

Accuracy assessment is essential for individual classifications if the classification data is to be useful in change detection (Owojori and Xie 2005). For the accuracy assessment of land cover maps extracted from satellite images, stratified random method was used to represent different land cover classes of the area. The accuracy was assessed by using 100 points, based on ground truth data and visual interpretation. The comparison of classification results and reference data was carried out statistically using error matrices. In addition, a non-parametric Kappa test was also performed to measure the extent of classification accuracy as it not only accounts for diagonal elements but for all the elements in the confusion matrix (Rosenfield and Fitzpatrick-Lins 1986).

### Land use/cover change detection

Post-classification change detection technique, performed in ArcGIS 10 was employed by the study. Post classification in urban environment has been effectively used by various researchers due to its efficiency in detecting the location, nature and rate of change (Hardin et al. 2007). Another technique used to obtain the changes in land cover/use during the specified time period was overlay procedure. A two-way cross-matrix obtained by the application of this was used to describe the key change types in the study area. Cross tabulation analysis was conducted in order to determine the quantitative conversions from a particular category to another land cover category and their corresponding area over the evaluated period on pixel to pixel basis. Thus, a new thematic layer was also produced from the two five-class maps, containing different combinations of ‘‘from–to’’ change classes.

## Results and discussion

**Fig. 3:**
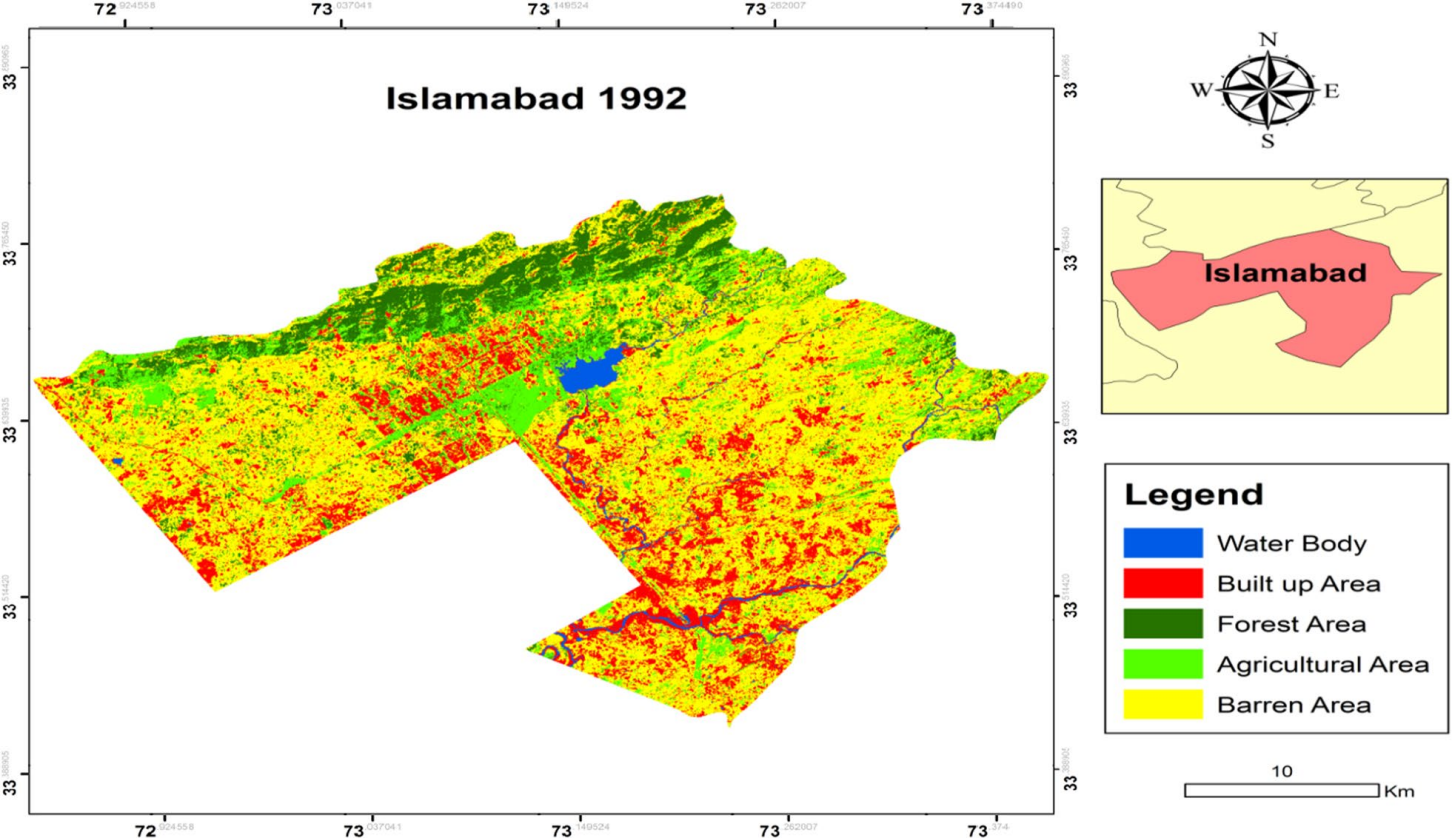
Classified land cover map of Islamabad in 1992

**Fig. 4:**
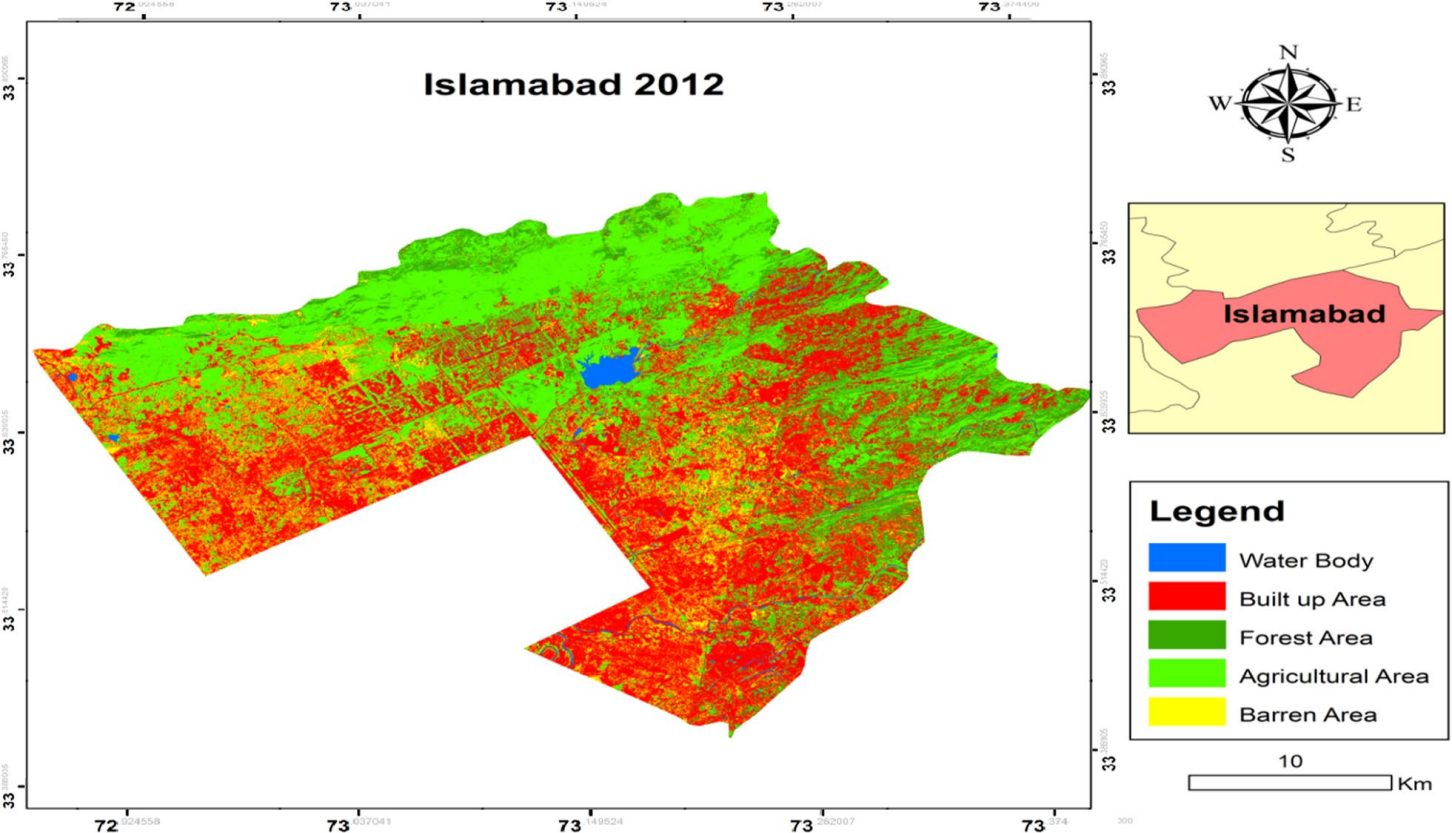
Classified land cover map of Islamabad in 2012

The resulting land use/cover maps of the two periods of 1992 and 2012 shown in Figs. 3 and 4 had an overall map accuracy of 89 % for both images by using error/confusion matrix. This is the commonly employed approach for evaluating per-pixel classification (Lu and Weng 2007). Kappa statistics/index was also computed for each classified map to measure the accuracy of the results. The resulting classification of land use/cover maps of the two periods had a Kappa statistics of 0.89 each. This was reasonably good overall accuracy and accepted for the subsequent analysis and change detection (Lea and Curtis 2010).

The results of the image classification showed that the total land area of Islamabad was 89,958 hectares (ha). Individual class area and change statistics for 1992 and 2012 are summarized in Table 3.

**Table 3:** Area statistics and percentage of the land use/cover units in 1992–2012

Land use and land cover categories	1992		2012		1992–2012	Annual change (%)
	Area (ha)	Area (%)	Area (ha)	Area (%)	Changed area (%)	
Water body	1416	1.57	1579	1.75	+11.5	+0.58
Built up area	16,281	18.09	51,039	56.73	+213.5	+10.67
Forest area	12,136	13.49	6138	6.82	−49.42	−2.47
Agricultural area	10,336	11.49	29,000	32.23	+180.6	+9.03
Barren area	49,789	55.35	1678	1.87	−96.63	−4.83

The percentage area of each class in 1992 and 2012 showed that Barren Area had the largest share in 1992 representing 55.35 % (49,789 ha) of the total LULC categories assigned. This class faced a major shift and it was reduced to 1.87 % (1678 ha) in 2012. The other class which faced decline during the study period was Forest Area. The area of this class in 1992 was 13.49 % (12,136 ha) of the total area and in 2012 it was reduced to 6.82 % (6138 ha). The other three classes faced an increase in the total share. The major increment was faced by Built up Area. Its share was increased from 18.09 % (16,281 ha) in 1992 to 56.73 % (51,039 ha) in 2012. The Agricultural Area was also increased from 11.49 % (10,336 ha) in 1992 to 32.23 % (29,000 ha) in 2012. The change in the Water class was not very significant though it was increased during the study period. The share in the total area was 1.57 % (1416 ha) in 1992 to 1.75 % (1579 ha) in 2012.

This study revealed that there was about more than 213 % increase of settlement area i.e. from 1992 to 2012 (Table 2). This value signified the dramatic land cover change on the category of built up surface exerting an incredible pressure on non-built up surfaces, in particular agricultural lands. Expansion of the already existing urban fabrics through rapid construction sites of residential units, commercial and industrial units and road networks and pavements port and leisure facilities and other impervious surfaces all combined together led to continuous expansion of built up surfaces in the different corners of the city. Chigbu et al. (2011) focused on the analysis of land use and cover changes in Aba Urban area, Nigeria by using medium satellite imageries for change detection. The results revealed that built up area was increased from 21.7 to 36.5 %. Another study of Tahir et al. (2013) on LULCC in Mekelle city, Ethiopia showed a positive change of 200 % in urban area.

The urban changes may be associated with population growth as well as industrial development during this period. Simultaneously, a close relationship of spatial urban expansion was shown with the geometric center of a city and distance from major roads, indicating that most significant drive to urban expansion was road. Deforestation and habitat loss were the major impacts of urbanization on the environment along Islamabad Highway. In this study, it was found that there is approximately 49 % decrease in forest cover since last 20 years i.e. from 1992 to 2012. One of the main reasons for the loss of forest to sparse vegetation can be explained by the immense damage caused by wild and accidental fires during the summers, when there is no rain for months and temperature soars up 45 °C and hundreds of hectares turn to cinders.

Conversion of dense forest to agricultural land settlement was also significant. The given data specifically state that the increase in deforestation was mainly due to increase in agricultural use of land but some of the forest areas were shifted to different gardens in the region. If deforestation continues the area is bound to face the negative impact of soil erosion, high temperature and dust storm (Ellis et al. 1994). These negative impacts would further lead to climatic changes and ripple effect would help in an increase of global warming in the future (Siddiqui 1991). Forest that remained intact during the study period were 50 % whereas 8 % of the forest land degraded indicating the trend towards the deforestation of forest to sparse vegetation especially along the western margins where most of the reserved forest suffered severe damage. Results indicated that the dense forest present during 1992 along the western margin had been completely changed to sparse vegetation by 2012 due to stone quarries, cultivation, wood cutting for fuel and fodder consumption by the villagers and the facto cement industry which borders the western periphery. Reports say that the continuous forest destruction in the city is causing a significant loss. The wood biomass declining rate is the second highest in the world and ranges from 4 to 6 % per year (UNEP 2004). Tripathi and Kumar (2012) analyzed the LULC dynamics in Takula Block (Uttarakand) by using modern geospatial techniques of remote sensing and GIS and the results revealed that forest decreased by 6.28 % from 1999 to 2005. Similarly, Reis (2008) investigated the land use and land cover changes in Rize, Turkey by using remote sensing and GIS. The LULCC were analyzed by change detection comparison and the results indicated that the agricultural land increased by 36.2 % from 1973 to 2002.

The result of classified maps also indicated the increasing rate of Water class share in total area of the city. Water bodies covered only 1416 ha of the study area land in 1992 and increased to 1579 ha in 2012. This fluctuation may be due to the rain fall in the Monsoon Season. Particularly, in this period, a huge volume of Rawal Lake has declined i.e. 594–478 ha during 20 years. The total area of Rawal Lake has shown a significant change and it accounted for a percentage of 19.5 %. Change detection analysis of Rawal watershed by Butt et al. (2015) also reported similar results. The watershed is confronting problems of rapid deforestation and urbanization resulting in gradual land use change. The population growth and increasing number of housing colonies in the catchment area of Rawal Lake are adversely affecting the water regime coming into lake. The activities, like cutting of trees due to intensive use for household needs and high market value (heating, cooking timber etc.), forest disease and ineffective forest management etc. are accelerating the rate of deforestation in the watershed area (IUCN 2005; Tanvir et al. 2006).

The area under vacant land decreased because of increasing population pressure in the core area compelling rich people to move to these vacant lands. This has resulted into the emergence of residential colonies in the outskirts of the city. The Barren class recorded negative change over the years under study (Table 2). The results of this study disclosed that the area decreased from 1992 to 2012 and the change was accounted for a percentage of 96.63 % in 20 years’ time period. Tahir et al. (2013) evaluated the LULCC in Mekelle city, Ethopia by using remote sensing and GIS. A negative change of 92.86 % was recorded in barren land and all farm lads available in the area were converted into other feature.

**Fig. 5:**
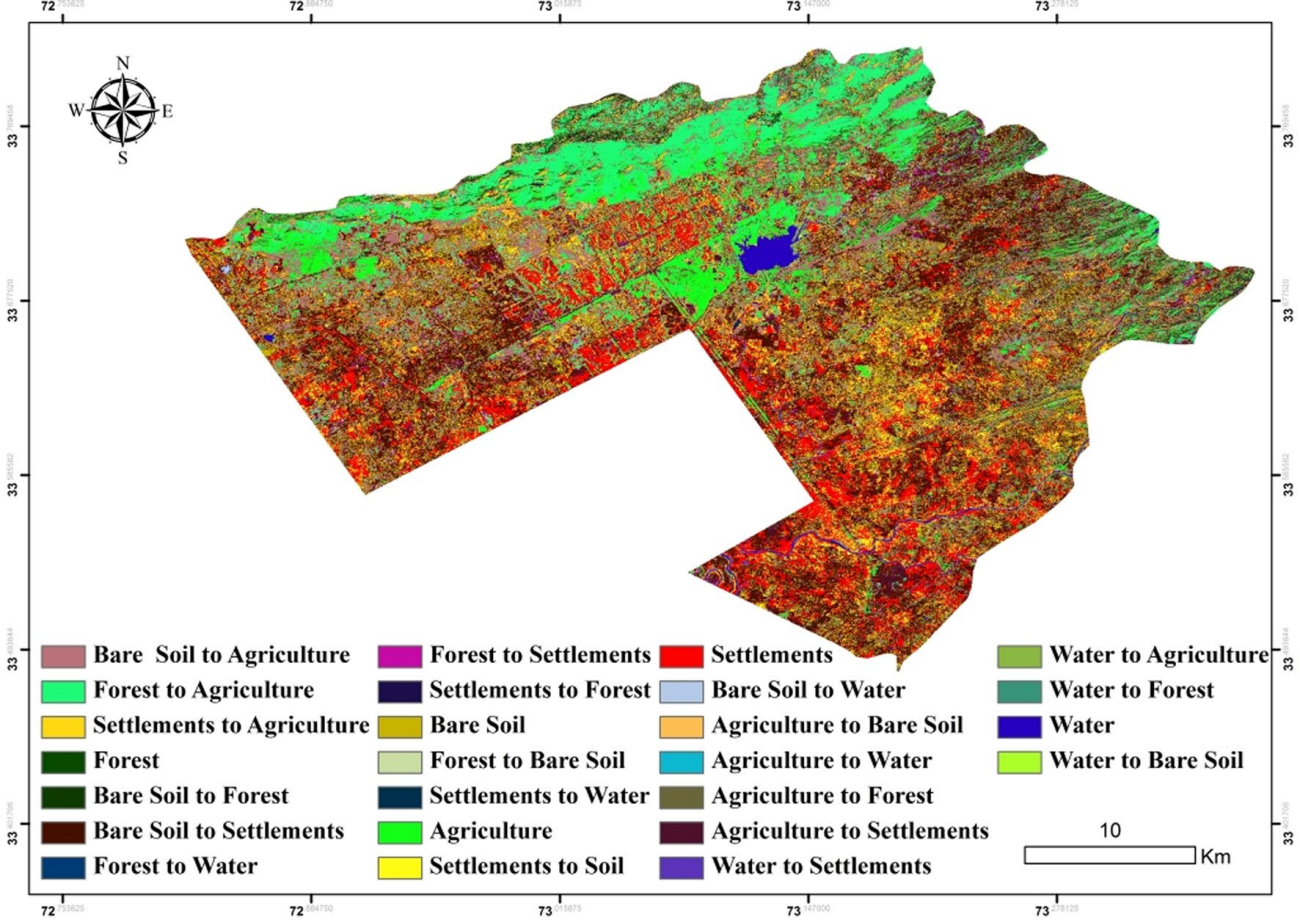
Major land use conversion in Islamabad from 1992 to 2012

Post-classification comparison of the detected change was carried out to produce change maps by using GIS, to comprehend the spatial patterns of change among years. The overlay of LULC maps was to produce the change map (Fig. 5) and the ‘from-to’ information given in Table 4 shows that the major observed change during the period of 20 years was from forest and bare soil/rock to agriculture and settlements.

**Table 4:** Major land use/cover conversions from 1992 to 2012

From class	To class	1992–2012 Area (ha)
Agriculture	Bare soil/rocks	1024.97
	Settlements	1831.23
	Forest	1610.70
	Water	122.12
Bare soil/rocks	Settlements	20,733.14
	Agriculture	12,443.91
	Forest	2705.05
	Water	390.62
Settlements	Bare soil/rocks	3867.04
	Agriculture	3092.5
	Forest	811.96
	Water	350.23
Forest	Agriculture	8211.17
	Settlements	1791.54
	Bare soil/rocks	1359.26
	Water	53.22
Water	Agriculture	197.02
	Settlements	371.85
	Forest	40.24
	Bare soil/rocks	79.64

### Driving forces

LULC changes of Islamabad are governed by a combination of environmental, geographical and socio-economic factors. Although the primary reason for rapid urbanization is population growth, the contribution of other causes such as environmental factors and economic development also need to be assessed. For the evaluation of underlying mechanisms of the changes in LULC, a regression analysis was performed of categories using selected environmental and socio-economic variables (GDP, population, temperature and precipitation), and the results are presented in Table 5. Socio-economic data, for instance GDP and population values were obtained from the yearly and decadal tables of the Pakistan Bureau of Statistics (Formerly Federal Bureau of Statistics).

**Table 5:** Regression analysis for land use/cover change and underlying factors

	R	R^2^	Adjusted R^2^	SE of estimate
Water body	0.934^a^	0.873	0.746	3.29
Forest area	0.986^a^	0.972	0.944	5.91
Agricultural area	0.998^a^	0.996	0.991	1.08
Built-up area	0.995^a^	0.991	0.982	2.81
Barren area	0.998^a^	0.996	0.993	6.33

^a^Predictors: (constant), population, GDP, temperature, rainfall

The regression analysis of land use parameters in relation to climatic variables, population and economic factors strengthened the influence and role of all these factors in land use conversion pattern in the study area. The coefficient of determination of 0.873, 0.972, 0.996, 0.991 and 0.996 computed for water body, forest area, agriculture area, built-up area and barren area respectively revealed that 87, 97 and 99 % of variance or change in the land use classes in the study area during the specified time period can be explained by the selected underlying factors.

The census data indicated the rapid population growth during the study period (Fig. 1). The rapid population growth in urban areas was mainly resulted from migration of rural to urban areas. This increase in population had a plausible effect of increase in pressure on the limited resource-base, and significantly contributed to the expansion of urban land by deforestation and infilling of low-lying areas.

Urban growth may have positive or negative impacts on environment but unplanned growth of urban areas always has negative effects. Environmental problems associated with urban growth tend to be analogous in both developing and developed countries. Urban settlements continue to increase on daily basis so the activities such as construction of buildings, parking lots and roads act as waterproof for the city surface. However, the land has drastically and irreversibly changed from its original state as the conversion from natural landscape to an urban area is an irreversible process. If there is further expansion in urban land it will be a destruction cause of a lot of more precious habitats. The urbanization process operating in the fringe has given rise to typical land use associations where there is side by side development of dynamic and contemporary land use pattern. Serious land use problems such as agricultural land losses and unauthorized urban sprawl have been emerged due to the emergence of this rural urban fringe zone with its complex problems of adjustments in between different ways of life in rural and urban areas.

Economic development of Islamabad is another factor contributing to rapid urbanization. The GDP of Islamabad in 1992 was approximately 0.49 billion US dollars, 0.72 billion US dollars in 2002. Currently, the GDP of the city is 2.11 billion US dollars and share of city in national economy is 1 % (www.dawn.com/news/844412/economics-and-extremism). The industrialization and economic development has led to higher urbanization rates. This economic activity has also resulted in influx of large number of rural immigrants during the study period.

**Fig. 6:**
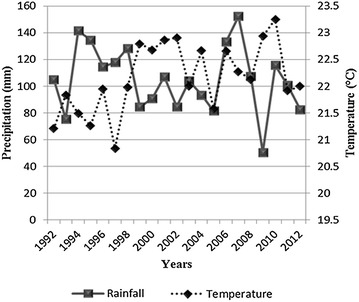
Change in temperature and precipitation of Islamabad from 1992 to 2012

One of the most significant ways that global climate change is predicted to affect economic activity is through its effect on agriculture, since temperature and precipitation are direct inputs to agricultural productions. Climate change has been postulated to affect forest nutrient cycles, agricultural productivity and other processes of ecological significance. For instance, changes in precipitation will cause hydrological fluxes of nutrients to change and could cause changes in productivity, decomposition and nutrient uptake. Similarly increases in temperature could also result in changes in hydrologic fluxes, decomposition, accelerated physiological development, resulting in reduced yield and hastened maturation (Johnson et al. 2000; Bonan 2008). Rainfall is the most significant factor in continuous destruction of forests and agricultural lands. In terms of precipitation, higher rainfalls could enhance growing period duration. In some areas of Pakistan high degree of precipitation has enabled higher degree of production and provided more water for irrigation. However, contrary consequences were observed in some regions due to loss of fertile soils with intense flooding caused by high average rainfalls or hindrance in drying and storage of crops. On the other hand, the declining rate of woody biomass is the second highest in the world. It ranges 4–6 % per year (UNEP 2004). This decline in forest area has been linked with ever changing climatic conditions that have been intensified over the years as a consequence of natural and man-made processes. The regression analysis revealed the impact of temperature and rainfall on the forest area as the average temperature has increased and the precipitation has decreased over the years (Fig. 6).

According to the IPCC, an increase in the average global temperature probably leads to changes in atmospheric moisture and precipitation. The impacts of the temperature rises include more intensive rainfall, floods, drying of some rivers in the dry season which were up till now perennial rivers, unpredictable weather such as late start of the rainfall season and or shorter rainy season and frequent events of drought due to low levels of water in the dams. All these are evidently impacts of climate change in Islamabad. Planners across many sectors will confront the challenge of a changing climate. They will likely adopt a variety of adaptation practices, designed to plan sustainable urbanization, better conserve water supplies, forest and agricultural land and develop alternative strategies for their management.

## Conclusion

In the present study, assessment of LULC and their change detection were carried out using digital image processing techniques. Analysis revealed that urban areas, agricultural areas and water increased during 1992–2012 resulting in substantial reduction of forest area and barren land. The increase in the water was insignificant. However, the major drinking water reservoir of the city faced a decline by 19.5 % during the study period. The conversion of forest and barren land to urban land has caused varied and extensive environmental degradation in the study area and the major negative outcomes associated with the rapid urban development are the growths of slums. Major driving forces of urban land expansion are population growth and economic development.
